# Cost-Effectiveness of Treating Upper Limb Spasticity Due to Stroke with Botulinum Toxin Type A: Results from the Botulinum Toxin for the Upper Limb after Stroke (BoTULS) Trial 

**DOI:** 10.3390/toxins4121415

**Published:** 2012-11-27

**Authors:** Phil Shackley, Lisa Shaw, Christopher Price, Frederike van Wijck, Michael Barnes, Laura Graham, Gary A. Ford, Nick Steen, Helen Rodgers

**Affiliations:** 1 School of Health and Related Research, University of Sheffield, Regent Court, 30 Regent Street, Sheffield S1 4DA, UK; 2 Institute for Ageing and Health, Newcastle University, Newcastle upon Tyne NE4 5PL, UK; Email: lisa.shaw@ncl.ac.uk (L.S.); c.i.m.price@ncl.ac.uk (C.P.); gary.ford@ncl.ac.uk (G.A.F.); helen.rodgers@ncl.ac.uk (H.R.); 3 Institute for Applied Health Research, School of Health and Life Sciences, Glasgow Caledonian University, Cowcaddens Road, Glasgow G4 0BA, UK; Email: frederike.vanwijck@gcu.ac.uk; 4 International Centre for Neurorehabilitation, Walkergate Park, Benfield Road, Newcastle upon Tyne NE6 4QD, UK; Email: m.p.barnes@btinternet.com (M.B.); laura.graham@ntw.nhs.uk (L.G.); 5 Institute of Health and Society, Newcastle University, Newcastle upon Tyne NE2 4AA, UK; Email: nick.steen@ncl.ac.uk

**Keywords:** botulinum toxin type A, stroke, upper limb spasticity, cost-effectiveness

## Abstract

Stroke imposes significant burdens on health services and society, and as such there is a growing need to assess the cost-effectiveness of stroke treatment to ensure maximum benefit is derived from limited resources. This study compared the cost-effectiveness of treating post-stroke upper limb spasticity with botulinum toxin type A plus an upper limb therapy programme against the therapy programme alone. Data on resource use and health outcomes were prospectively collected for 333 patients with post-stroke upper limb spasticity taking part in a randomized trial and combined to estimate the incremental cost per quality adjusted life year (QALY) gained of botulinum toxin type A plus therapy relative to therapy alone. The base case incremental cost-effectiveness ratio (ICER) of botulinum toxin type A plus therapy was £93,500 per QALY gained. The probability of botulinum toxin type A plus therapy being cost-effective at the England and Wales cost-effectiveness threshold value of £20,000 per QALY was 0.36. The point estimates of the ICER remained above £20,000 per QALY for a range of sensitivity analyses, and the probability of botulinum toxin type A plus therapy being cost-effective at the threshold value did not exceed 0.39, regardless of the assumptions made.

## 1. Introduction

Stroke is a major cause of mortality and morbidity and imposes a significant burden on both health services and society [1,2,3]. In the United Kingdom (UK) it is estimated that the annual direct costs of stroke are approximately £4 billion, which constitutes around 5.5% of the total UK expenditure on health care [3]. If the costs of lost productivity and informal care are taken into account, the total annual societal costs of stroke are estimated to be around £9 billion [3]. In England, over 900,000 people are living with the consequences of stroke, 300,000 of whom are moderately or severely disabled [4]. As the proportion of older people in society increases, so the burden of stroke is likely to grow.

Upper limb spasticity after stroke is an important clinical problem and its identification and treatment are key components of stroke rehabilitation [5]. Upper limb spasticity may cause deformity, reduced function and pain [6]. Botulinum toxin type A, which when given by intramuscular injection causes temporary local muscle paresis by blocking neuromuscular transmission [7], has become an established treatment for spasticity due to stroke. Randomised controlled trials have shown that botulinum toxin reduces muscle tone [8] and improves the performance of basic upper limb functional tasks such as hand opening for cleaning and ease of dressing [9,10,11]. However, the impact on active upper limb function (e.g., reaching and grasping) and the efficacy of repeated treatment is less clear. 

The BoTULS trial was a pragmatic multi-centre randomised controlled trial to evaluate the clinical and cost-effectiveness of botulinum toxin type A plus an upper limb therapy programme in the treatment of post stroke upper limb spasticity. The clinical results indicated that botulinum toxin type A did not improve active upper limb function (as measured by the Action Research Arm Test (ARAT)), but that there may be benefits in terms of decreased muscle tone, improved upper limb strength, improved ease of performance of basic upper limb functional activities and reduction in pain [12]. This article describes the results of the cost-effectiveness analysis.

## 2. Methods

In this multi-centre trial, 333 adults with spasticity and reduced upper limb function due to stroke greater than one month previously were randomised to receive botulinum toxin type A (Dysport^®^) plus a four week upper limb therapy programme comprising of one hour of therapy twice weekly (*n* = 170), or the upper limb therapy programme alone (*n *= 163). Repeat botulinum toxin type A and/or therapy was available at three, six and nine months. Patients were assessed at baseline and at one, three, and 12 months. Study methods have been reported in detail elsewhere [12]. The trial received ethical and NHS approvals, and the study participants provided informed consent.

The economic evaluation follows the technology appraisal guidelines used by the National Institute for Health and Clinical Excellence (NICE) and as such adopts the perspective of the UK National Health Service and Social Services [13]. The time horizon for the analysis was three months from randomisation, with all costs reported in 2007 prices.

**Table 1:** Breakdown of resource use and corresponding unit cost data.

Resource	Unit cost (2007 prices)	Source of unit cost data
Upper limb therapy and botulinum toxin type A		
therapist	£40 per session ^a^	Reference14
botulinum toxin type A	£153.21 per 500 unit vial	Reference 15
Other antispasticity medication		
gabapentin	£96.73 per month	Reference 15
baclofen	£9.13 per month	Reference 15
tizanidine	£74.83 per month	Reference 15
dantrolene	33.76 per month	Reference 15
methocarbamol	7.60 per month	Reference 15
Other health care & social services		
day hospital	£83 per place per day	Reference 14
home care services	£19 per contact ^b^	Reference 14
private home help	£11.33 per contact ^b^	Reference 14
day centre	£147 per attendance	Reference 14
meals on wheels	£3.63 per meal	Reference 14
laundry service	£3 per wash	Assumption ^c^
GP	£36 per consultation	Reference 14
practice nurse	£9 per consultation	Reference 14
district nurse	£24 per home visit	Reference 14
health visitor	£36 per home visit	Reference 14
physiotherapist	£40 per contact ^b^	Reference 14
occupational therapist	£40 per contact ^b^	Reference 14
speech and language therapist	£40 per contact ^b^	Reference 14
dietician	£32 per contact ^b^	Reference 14
chiropodist	£18 per contact ^b^	Reference 14
social worker	£34 per contact ^b^	Reference 14
clinical psychologist	£67 per contact ^b^	Reference 14
continence advisor	£24 per home visit	Reference 14
bath attendant	£11.33 per contact ^b^	Assumption ^d^
orthotist	£40.00 per contact ^b^	Assumption ^e^

^a^ Assumes therapist administers injection; ^b^Assumes one hour per contact; ^c^ Based on local council fees of £3 per wash; ^d^ Assumed same rate as private home help; ^e^ Assumed Agenda for Change Band 5.

Participants’ use of resources was categorised under five general headings: (1) botulinum toxin type A; (2) upper limb therapy sessions provided by chartered physiotherapists; (3) other antispasticity medication; (4) management of adverse events attributable to botulinum toxin type A and/or upper limb therapy requiring a hospital contact; and (5) other health care and social services resource use. Patients’ use of these resources was recorded throughout the trial using a combination of case record forms, adverse event monitoring forms and participant responses to specific resource use questions in the participant assessment questionnaires. Where data were missing, resource use was inferred. Unit cost data were obtained from national sources [14,15]. All unit costs are reported in Table 1 and are in pounds sterling.

Participant health related quality of life was assessed using the EuroQol (EQ-5D) [16] which was included in the participant assessment questionnaires. Participant responses to the EQ-5D questionnaire were converted to health state utility values using the UK tariff values [17].These values were then multiplied by duration in each health state to estimate quality adjusted life years (QALYs). QALYs were estimated using an area under the curve (AUC) approach [18] using participant responses at baseline and three months to map out the curve.

In order to assess the relative cost-effectiveness of botulinum toxin type A plus upper limb therapy relative to upper limb therapy alone, data on cost and outcome were brought together to estimate an incremental cost-effectiveness ratio (ICER), specifically, the incremental cost per QALY gained. Uncertainty surrounding the point estimate of the ICER was taken into account through the approach of non-parametric bootstrapping and the construction of a cost-effectiveness acceptability curve (CEAC), which summarises the evidence in support of botulinum toxin type A plus therapy being cost-effective for a range of threshold values of societal willingness to pay for a QALY [19].

In addition to addressing the uncertainty surrounding the point estimate of the ICER, sensitivity analysis was undertaken to investigate the impact on the results of making different assumptions and varying key parameters. As part of the sensitivity analysis, in order to take into account missing data, an additional set of analyses were carried out using the technique of multiple imputation in which missing data were imputed using the NORM package [20]. Five datasets were imputed using age, sex, place of residence, Barthel ADL score and time between stroke and randomisation as explanatory variables. A point estimate of the ICER and accompanying CEAC for botulinum toxin type A plus therapy were estimated.

## 3. Results

The base case analysis used data from 283/333 (85%) participants who provided EQ-5D responses at baseline and three months, of whom 150 were in the botulinum toxin type A plus therapy group and 133 were in the therapy alone group. The randomisation groups were well matched at baseline with regard to demography, stroke characteristics, comorbidity, upper limb problems, and quality of life (including EQ-5D scores).

### 3.1. Resource Use and Costs

There was no significant difference in the mean number of upper limb therapy sessions received by participants in the botulinum toxin type A plus therapy and therapy alone groups (7.64 *versus* 7.56 respectively; 95% CI of the difference = −0.281 to 0.127). The numbers of participants taking other antispasticity drugs were 38 in the botulinum toxin type A plus therapy group and 31 in the therapy alone group, with the difference between the groups not being significant (odds ratio (OR) = 1.116; 95% CI = 0.647 to 1.925). While there were cases of participants encountering hospital services as a consequence of their initial stroke, clinical review showed that none of the hospital contacts were attributable to either therapy or botulinum toxin type A. As a result, hospital resource use due to adverse events does not feature in the cost-effectiveness analysis.

A breakdown of other health care and social services resource use among participants is presented in Table 2. With respect to the proportion of participants in each group reporting a contact, there were significantly more practice nurse contacts (OR = 1.878; 95% CI = 1.083 to 3.255) and social worker contacts (OR = 1.917; 95% CI = 1.051 to 3.497) in the botulinum toxin type A plus therapy group.

The only significant difference in the average number of contacts among participants reporting a contact was with respect to day hospital contacts, with participants in the therapy alone group having significantly more contacts on average (9.4 *versus* 3.1 respectively; 95% CI of the difference = 2.42 to 10.18).

**Table 2:** Breakdown of other health care and social services resource use.

Item of resource use	Mean (SD) number of contacts among patients reporting a contact (n)				Mean difference in number of contacts (95% CI of difference)
	Therapy alone		Therapy plus botulinum toxin		
	Mean (SD)	*n*	Mean (SD)	*n*	
day hospital	9.4 (9.0)	25	3.1 (2.7)	21	6.3 (2.4 to 10.2)
home care services	87.5 (75.7)	45	114.6 (76.5)	56	−27.1 (−57.3 to 3.2)
private home help	32.7 (53.5)	17	38.9 (60.1)	16	−6.2 (−46.6 to 34.1)
day centre	16.3 (8.3)	25	14.1 (10.3)	24	2.2 (−3.1 to 7.6)
meals on wheels	6.0	1	56.0 (38.6)	3	−50.0 (−241.7 to 141.7)
laundry service	14.7 (18.5)	3	18.0 (11.1)	3	−3.3 (−38.0 to 31.3)
GP	2.8 (1.4)	60	2.8 (1.9)	86	0.0 (−0.6 to 0.6)
practice nurse	2.6 (1.8)	24	2.9 (2.9)	44	−0.3 (−1.6 to 1.0)
district nurse	8.3 (14.6)	27	4.0 (5.0)	30	4.3 (−1.8 to 10.2)
health visitor *	-	1	1.0	1	-
physiotherapist	13.3 (12.7)	74	12.0 (10.5)	87	1.3 (−2.46 to 4.8)
occupational therapist	8.2 (8.8)	26	8.5 (9.1)	38	−0.3 (−4.9 to 4.2)
speech and language therapist	4.6 (5.3)	28	8.9 (9.7)	20	−4.3 (−9.2 to 0.6)
dietician	2.2 (1.2)	6	13.0 (15.6)	2	−10.8 (−149.5 to 127.8)
chiropodist	2.0 (1.2)	24	2.3 (1.1)	40	−0.3 (−0.9 to 0.3)
social worker	2.8 (1.7)	17	2.5 (2.0)	35	0.3 (−0.8 to 1.5)
clinical psychologist	3.1 (2.5)	8	2.5 (1.3)	4	0.6 (−2.4 to 3.7)
continence advisor	1.8 (1.0)	4	1.0	1	0.8 (−2.7 to 4.2)
bath attendant	19.5 (18.9)	13	18.2 (11.4)	13	1.3 (−11.3 to 14.0)
orthotist	1.00	1	2.7 (1.2)	3	−1.7 (−7.4 to 4.1)

* The one patient in the therapy alone group who reported a health visitor contact did not indicate the number of contacts.

Table 3 shows the contribution of botulinum toxin type A costs, upper limb therapy costs, other antispasticity medication costs, and other health care and social services costs to the overall mean cost per participant. The overall mean cost per participant was higher in the botulinum toxin type A plus therapy group, although the difference was not significant. There were also no significant differences between the groups with respect to upper limb therapy costs, antispasticity medication costs and other health care and social services costs. The biggest contributor to total costs for both groups was the cost of other health care and social services contacts, accounting for 81% in the therapy alone group and 77% in the botulinum toxin type A plus therapy group. The mean difference in costs indicates that even if the cost of botulinum toxin type A were zero, there would still a positive net cost associated with the botulinum toxin type A group. This is largely attributable to the botulinum toxin type A group having higher other health care and social services costs.

**Table 3:** The contribution of botulinum toxin type A costs, upper limb therapy costs, other antispasticity medication costs, and other health care and social services costs to overall mean cost per participant.

Breakdown of overall mean cost per participant	Mean (SD) cost per participant (£)		Mean difference in costs (£) (95% CI of difference)
	Therapy alone	Therapy plus botulinum toxin	
Overall	1796 (1944)	2170 (2007)	−374 (−837 to 90)
Botulinum toxin	3 ^a^ (23)	154 (28)	−151 (−157 to −145)
Upper limb therapy	300 (45)	303 (41)	−3 (−13 to 7)
Antispasticity medication	37 (93)	38 (90)	−1 (−22 to 21)
Other ^b^	1456 (1923)	1675 (2001)	−219 (−679 to 242)

^a^ EQ-5D data were complete for 3 of 4 participants in the control group who received botulinum toxin; ^b^ Other health care and social services costs.

**Table 4:** Summary of cost-effectiveness results for botulinum toxin type A plus therapy for alternative scenarios.

Cost-effectiveness analysis scenario	IC (£)	IQ (QALYs)	ICER (£)	Probability botulinum toxin type A plus therapy is cost-effective at threshold ratio			
				£10 k	£20 k	£50 k	£100 k
Base case data ^a^	374	0.004	93,500	0.29	0.36	0.41	0.42
Complete EQ-5D data ^b^	482	0.007	68,857	0.29	0.34	0.40	0.43
Base case data and zero cost for botulinum toxin	223	0.004	55,750	0.31	0.39	0.42	0.43
Base case data and best-worst QALY assumptions	374	0.006	62,333	0.29	0.35	0.43	0.45
Missing data imputed using multiple imputation	430	0.005	86,000	0.34	0.39	0.42	0.46

IC = Incremental cost; IQ = Incremental QALYs; ICER = Incremental cost-effectiveness ratio; ^a^ EQ-5D responses at baseline and 3 months; ^b^ EQ-5D responses at baseline, 1 month and 3 months.

### 3.2. Base Case Cost-Effectiveness Analysis

**Figure 1:**
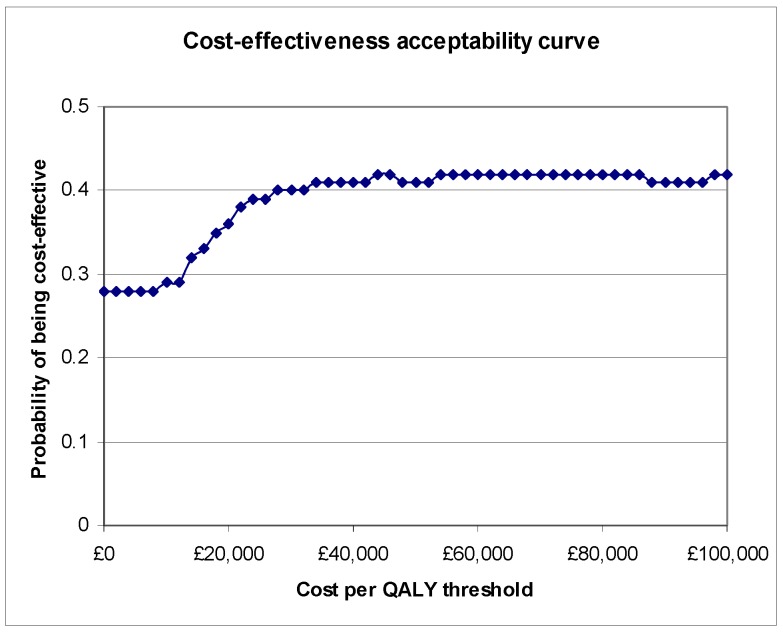
CEAC for botulinum toxin type A plus therapy relative to therapy alone.

The results of the base case cost-effectiveness analysis are summarised in the first row of Table 4. Compared to therapy alone, botulinum toxin type A plus therapy was associated with an incremental cost of £374 and an incremental QALY gain of 0.004. When combined, these data gave an ICER for botulinum toxin type A plus therapy of £93,500 per QALY gained. Bootstrapping this point estimate enabled the derivation of the CEAC for botulinum toxin type A plus therapy relative to therapy alone shown in Figure 1. The probabilities of botulinum toxin type A plus therapy being cost-effective at threshold ratios of £10,000, £20,000, £50,000 and £100,000 per QALY were 0.29, 0.36, 0.41 and 0.42 respectively.

### 3.3. Sensitivity Analysis

The impact on the results of the following sensitivity analyses were explored: re-running the analysis using data from participants with complete EQ-5D data at baseline and at one and three months; allowing the cost of botulinum toxin type A to fall to zero; a best-worst QALY analysis investigating the impact of alternative assumptions regarding the timing of health state changes; and re-running the analysis following multiple imputation of missing data. All the results are summarised in Table 4.

Re-running the analysis to include only those participants for whom complete EQ-5D data were available (248 respondents, of whom 116 were in the botulinum type plus therapy group and 132 were in the therapy alone group) had little impact on the results. The ICER for botulinum toxin type A plus therapy relative to therapy alone was £68,857 and the probabilities of its being cost-effective at threshold ratios of £10,000, £20,000, £50,000 and £100,000 per QALY were 0.29, 0.34, 0.40 and 0.43 respectively.

Re-running the base case analysis assuming a zero cost for botulinum toxin type A resulted in an ICER for botulinum toxin type A plus therapy of £55,750. The probabilities of its being cost-effective at threshold ratios of £10,000, £20,000, £50,000 and £100,000 per QALY were 0.31, 0.39, 0.42 and 0.43 respectively.

The AUC approach to estimating QALYs described above assumes that the rate of change in health status between any two points (in this case, EQ-5D tariff values) is linear. For example, if the baseline value is 0.5 and the three months value is 0.8, then it is assumed that after one month, the health state value is 0.6, and after two months it is 0.7. This is the method most commonly employed in AUC analyses in the literature [18]. However, many other assumptions could be made, each of which may have an impact on the results. In light of the relatively high ICERs associated with botulinum toxin type A plus therapy, it was decided to investigate the impact on the results of the timing of the health state changes favouring the use of botulinum toxin type A. Specifically, it was assumed in the botulinum toxin type A plus therapy group that participants moved into the health state value reported at three months almost immediately (approximately three days or 0.1 of a month) after baseline. This can be regarded as the best case outcome scenario for botulinum toxin type A. On the flip side, a worst case outcome scenario for the therapy alone group was defined whereby it was assumed that participants in this group remained in their baseline reported health state for 2.9 months, at which point they moved into the health state value reported at three months. The results of these assumptions were that botulinum toxin type A plus therapy had an ICER of £62,333 and the probabilities of its being cost-effective at threshold ratios of £10,000, £20,000, £50,000 and £100,000 per QALY were 0.29, 0.35, 0.43 and 0.45, respectively.

**Figure 2:**
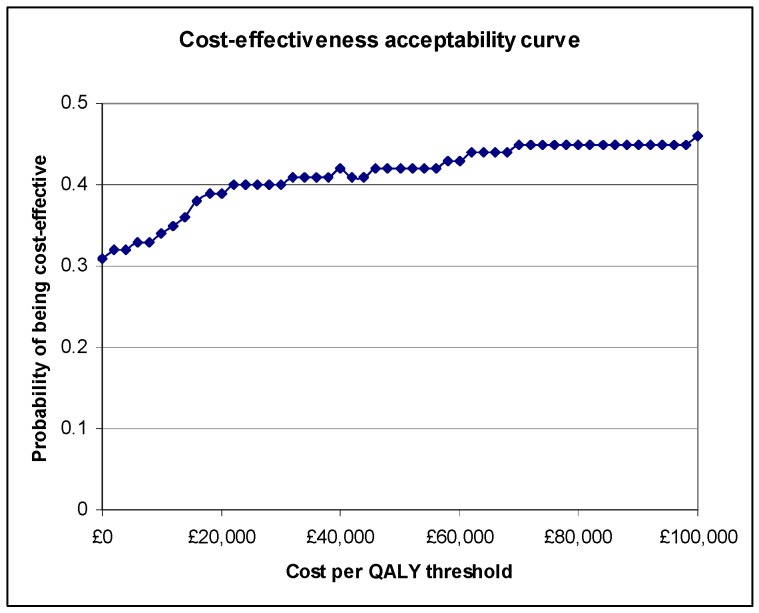
Cost-effectiveness acceptability curve (CEAC) for botulinum toxin type A plus therapy relative to therapy alone following multiple imputation of missing data.

Imputing missing data using multiple imputation resulted in botulinum toxin type A plus therapy having an ICER of £86,000. Figure 2 shows the CEAC for botulinum toxin type A plus therapy relative to therapy alone when missing data have been imputed. The probabilities of botulinum toxin type A plus therapy being cost-effective at threshold ratios of £10,000, £20,000, £50,000 and £100,000 per QALY were 0.34, 0.39, 0.42 and 0.46, respectively.

## 4. Discussion

The base case incremental cost-effectiveness ratio (ICER) for botulinum toxin type A plus therapy was £93,500, which is well in excess of the £20,000 threshold value used by NICE [21]. Estimation of the CEAC for botulinum toxin type A plus therapy indicated that there was only a 0.36 probability of its being cost-effective at a threshold ratio of £20,000 per QALY.

Sensitivity analysis using participants with complete EQ-5D data at three months produced an improved point estimate for the ICER of £68,857, but this was still over three times the NICE threshold value. The CEAC for these participants suggests that the probability of botulinum toxin type A plus therapy being cost-effective at £20,000 per QALY was actually lower than in the base case (0.34 *versus* 0.36 respectively).

Sensitivity analysis on the cost of botulinum toxin type A revealed that even if the cost of the drug were zero, the point estimate of the ICER still exceeded the NICE threshold value (£55,750 *versus* £20,000, respectively), and the probability of botulinum toxin type A plus therapy being cost-effective at £20,000 was little changed from the base case (0.39 *versus* 0.36 respectively).

Altering the assumptions regarding the timing of the health state changes following treatment so that they favoured botulinum toxin type A plus therapy (best-worst QALY analysis) resulted in a lower ICER than in the base case analysis (£62,333 *versus* £93,500, respectively). However, the probability that botulinum toxin plus therapy was cost-effective at £20,000 per QALY was actually lower than in the base case analysis (0.35 *versus* 0.36, respectively).

Imputing missing data had little impact on the results, with the use of multiple imputation resulting in an ICER of £86,000 for botulinum toxin plus therapy and a probability of 0.39 of its being cost-effective at £20,000 per QALY.

The study does have limitations. Arguably the main limitation was the relatively short time horizon over which the cost-effectiveness analysis was conducted. The rationale for adopting a three month time horizon rather than 12 months was the loss of participant responses due to a curtailment of 12 month follow up. The proportion of participants providing EQ-5D data at 12 months was 52.4%. This is considerably lower than the corresponding figures for baseline, one month and three months which were 100%, 83.7% and 85.2%, respectively.

One option would have been to impute the missing 12 month data, but this would have meant that even if the estimation of QALYs was restricted to the baseline and 12 month EQ-5D values only, data would have to be imputed for almost half the sample. If the intermediate EQ-5D values were also used in the QALY estimation, then data for more than half the sample would need to be imputed. Thus, a decision was made to conduct the analysis over three months where levels of missing data were much lower. This is not to say that there would not be benefits in extrapolating costs and outcomes over a longer time period. This could be done using economic modelling which would incorporate data from the trial and other relevant sources to estimate cost-effectiveness beyond the time horizon of the trial. While such an exercise was beyond the scope of the study reported here, it would be a potentially worthwhile piece of future research.

Another limitation was the need to infer resource use on occasions when data were not readily available. For example, for a number of patients it was necessary to make assumptions regarding length of time on specific antispasticity medications and the dosages taken. Assumptions also had to be made regarding the use of other health care and social services resources during the second month of the three month analysis period. This was due to resource use questions in the participant assessment questionnaires asking about resource use over the previous month only.

Another potential limitation arises from the possibility that the EQ-5D may not be sensitive enough to pick up all aspects of improvement associated with the interventions, such as botulinum toxin type A recipients having decreased muscle tone, improved upper limb strength, improved ease of performance of basic upper limb functional activities and reduction in pain. Use of a more sensitive condition-specific outcome measure in the economic analysis may have produced results more favourable to botulinum toxin type A. However, the use of condition-specific measures to make cross programme comparisons is controversial, and NICE currently recommends that health states are described using a standardised and validated generic instrument, with the EQ-5D being its measure of choice.

With respect to how our results compare with other cost-effectiveness analyses of botulinum toxin type A in this treatment context, to our knowledge only one other published study exists [22]. This study adopted a decision analytic approach to compare the cost-effectiveness of botulinum toxin type A injections with oral therapy in the treatment of post-stroke spasticity. It concluded that botulinum toxin type A is a cost-effective treatment for post-stroke spasticity, which is contrary to our findings. However, we believe that our study is methodologically superior for a number of reasons. Chief among these is the source of data on resource use and outcomes. Our study collected these data from actual patients as part of a prospective randomized trial using carefully designed and validated measures whereas the earlier study [22] relied exclusively on expert opinion. In addition, the measure of outcome used in the earlier study [22] was cost per successfully treated month which does not explicitly encompass quality of life impacts and is a much more limited measure of outcome than cost per QALY gained.

## 5. Conclusions

In conclusion, the results of this study indicated that even the lowest point estimate of the ICER for botulinum toxin type A plus therapy was over two and a half times the NICE cost-effectiveness threshold value (£55,750 *versus* £20,000, respectively), and the probability of its being cost-effective at the threshold value did not exceed 0.39, regardless of the assumptions made. Therefore, according to current criteria for cost-effectiveness in England and Wales [21], the economic analysis provides no evidence to suggest that botulinum toxin type A plus therapy is a cost-effective alternative to therapy alone in this patient group.
